# Supplementary material for: Antimicrobial peptide glatiramer acetate targets Pseudomonas aeruginosa lipopolysaccharides to breach membranes without altering lipopolysaccharide modification
Source: NPJ Antimicrob Resist. 2024 Feb 20;2:4. doi: 10.1038/s44259-024-00022-x (PMC11702655; doi:10.1038/s44259-024-00022-x)
Supplement: Supplementary file 1 — Supplementary Information [file 44259_2024_22_MOESM1_ESM.pdf]

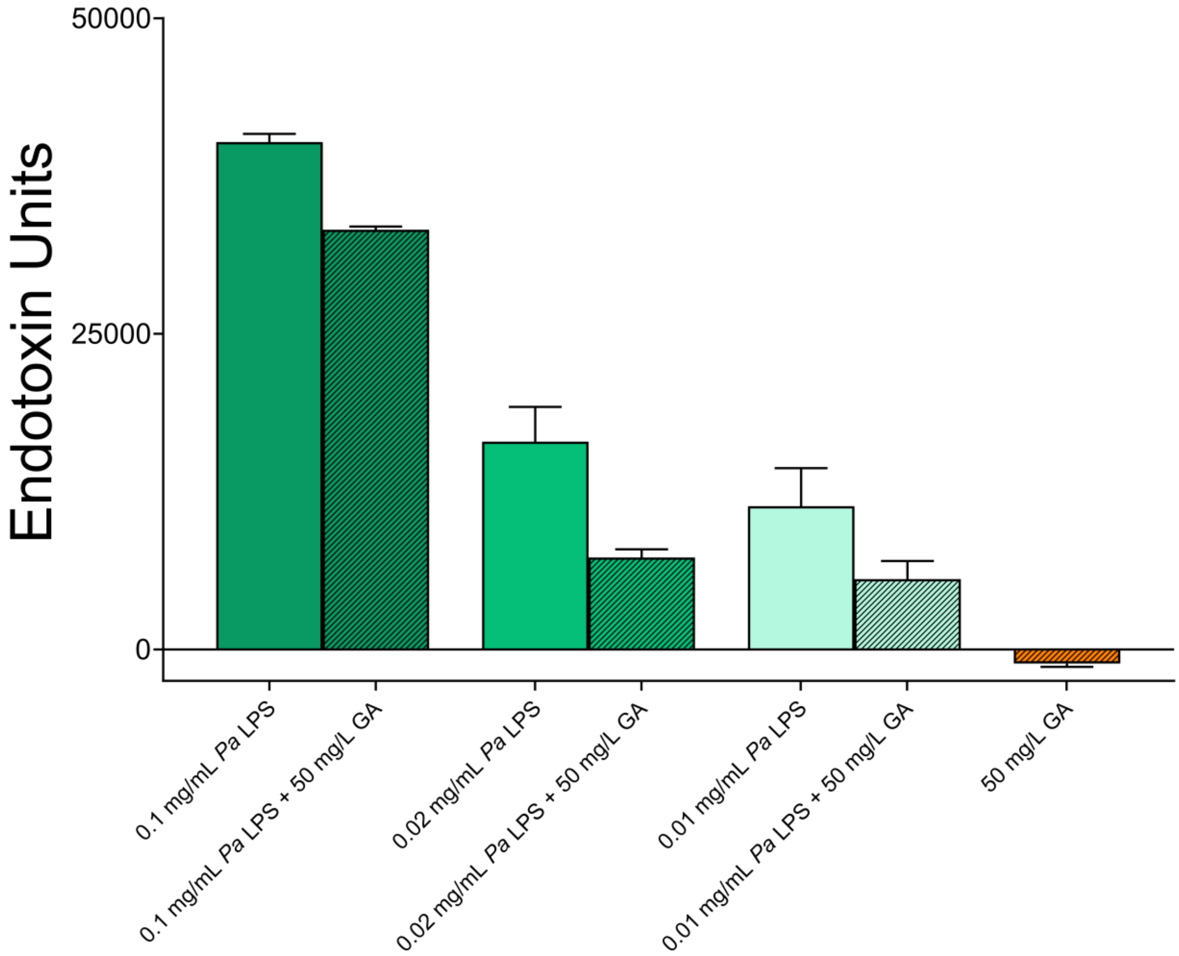

Supplementary Figure 1. Endotoxin Units of 0.1, 0.02 and 0.01 mg/mL P. aeruginosa LPS (without and with 50 mg/L GA), along with 50 mg/L GA alone, detected by limulus amoebocyte lyase (LAL) Endotoxin Quant Kit. Median with 95%CI. n = 3.

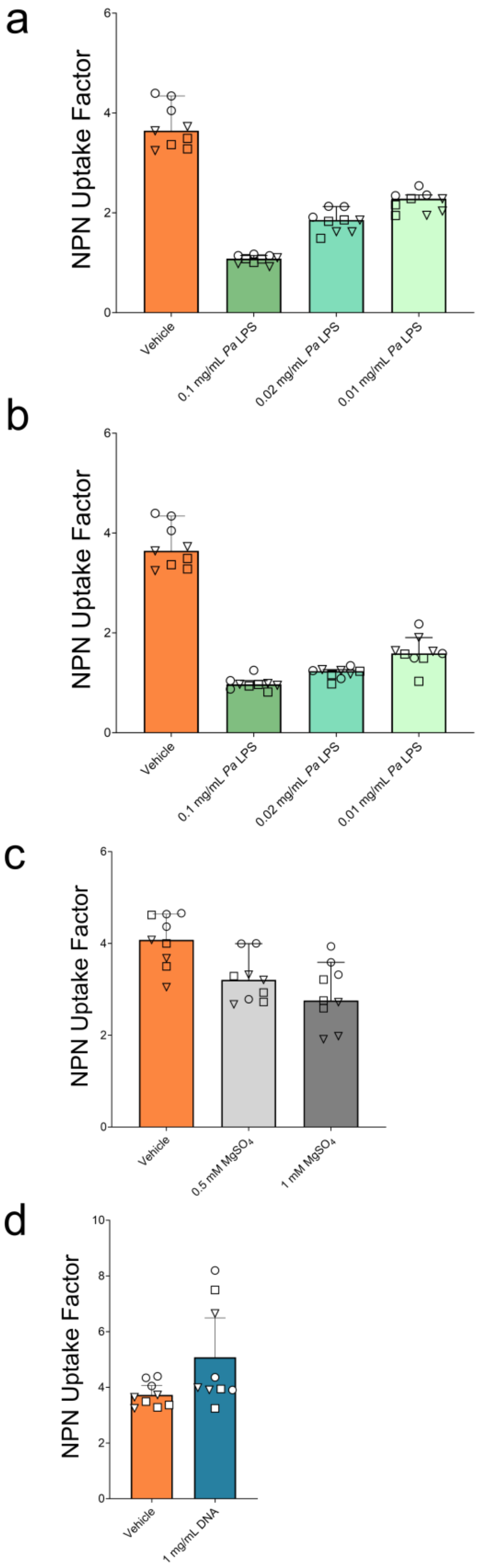

Supplementary Figure 2. Untransformed NPN Uptake Factor of ***a****. Pre-incubated LPS and GA.* ***b.*** *Background LPS with GA* ***c****. $MgSO_4$* ***d****. DNA. Medians with 95% CIs of biological replicates (n = 9) of P. aeruginosa PAO1 (○), PA14 (□) or PAK (△).*

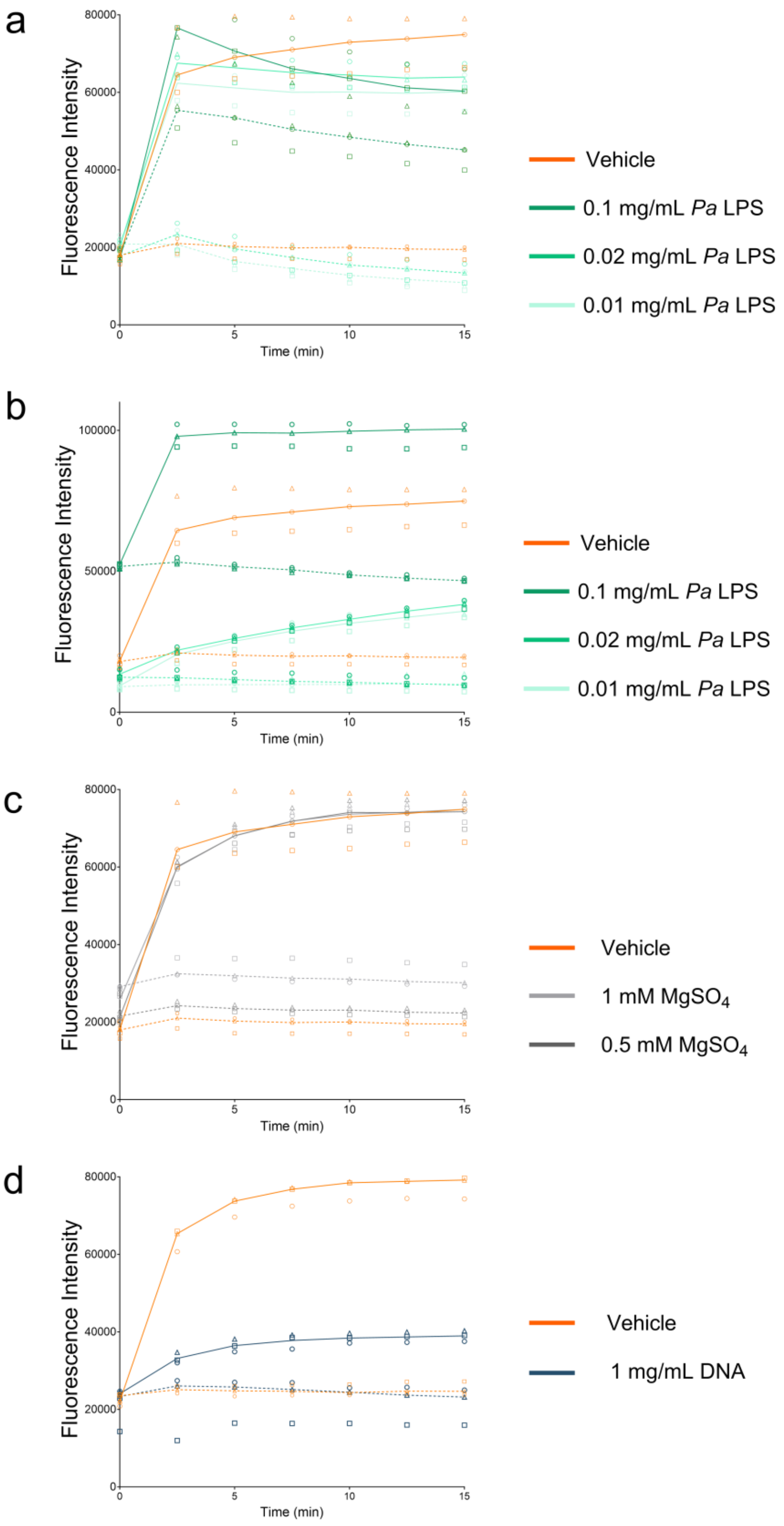

*Supplementary Figure 3. Release of DiSC$_3$(5) from the cytoplasmic membrane over 15mins after treatment with GA for **a**. Pre-incubated P. aeruginosa LPS and GA **b**. Background P. aeruginosa LPS **c**. $MgSO_4$ **d**. DNA. Solid Lines – 50 mg/L GA treated. Dotted Lines – Untreated. Median of type strains with points for each P. aeruginosa PAO1 (○), PA14 (□) or PAK (△).*

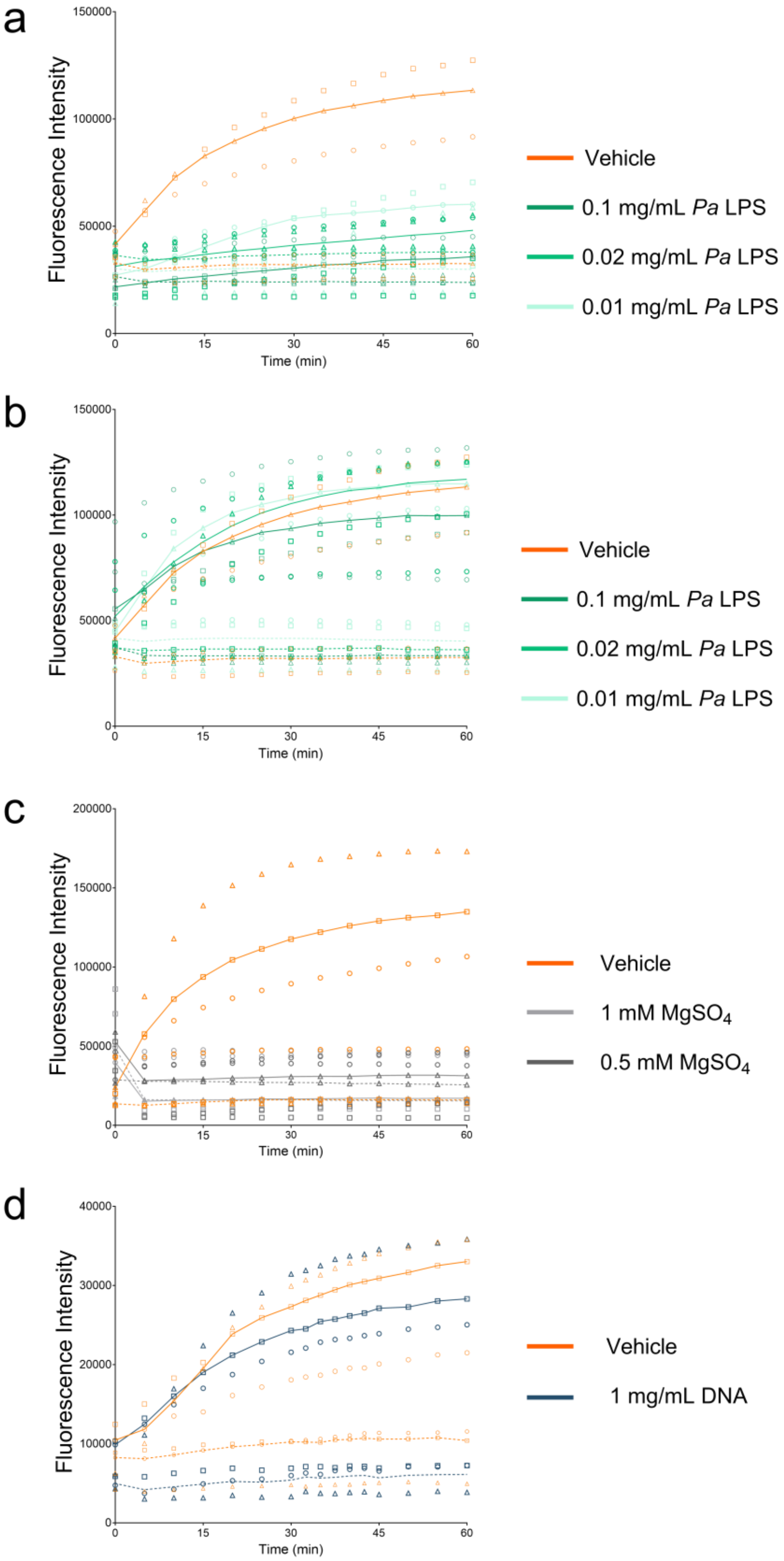

Supplementary Figure 4. Propidium iodide fluorescence over 60mins after treatment with GA for ***a****. Pre-incubated P. aeruginosa LPS and GA* ***b****. Background P. aeruginosa LPS* ***c****. $MgSO_4$* ***d****. DNA. Solid Lines – 50 mg/L GA treated. Dotted Lines – Untreated. Median of type strains with points for each P. aeruginosa PAO1 (○), PA14 (□) or PAK (△).*

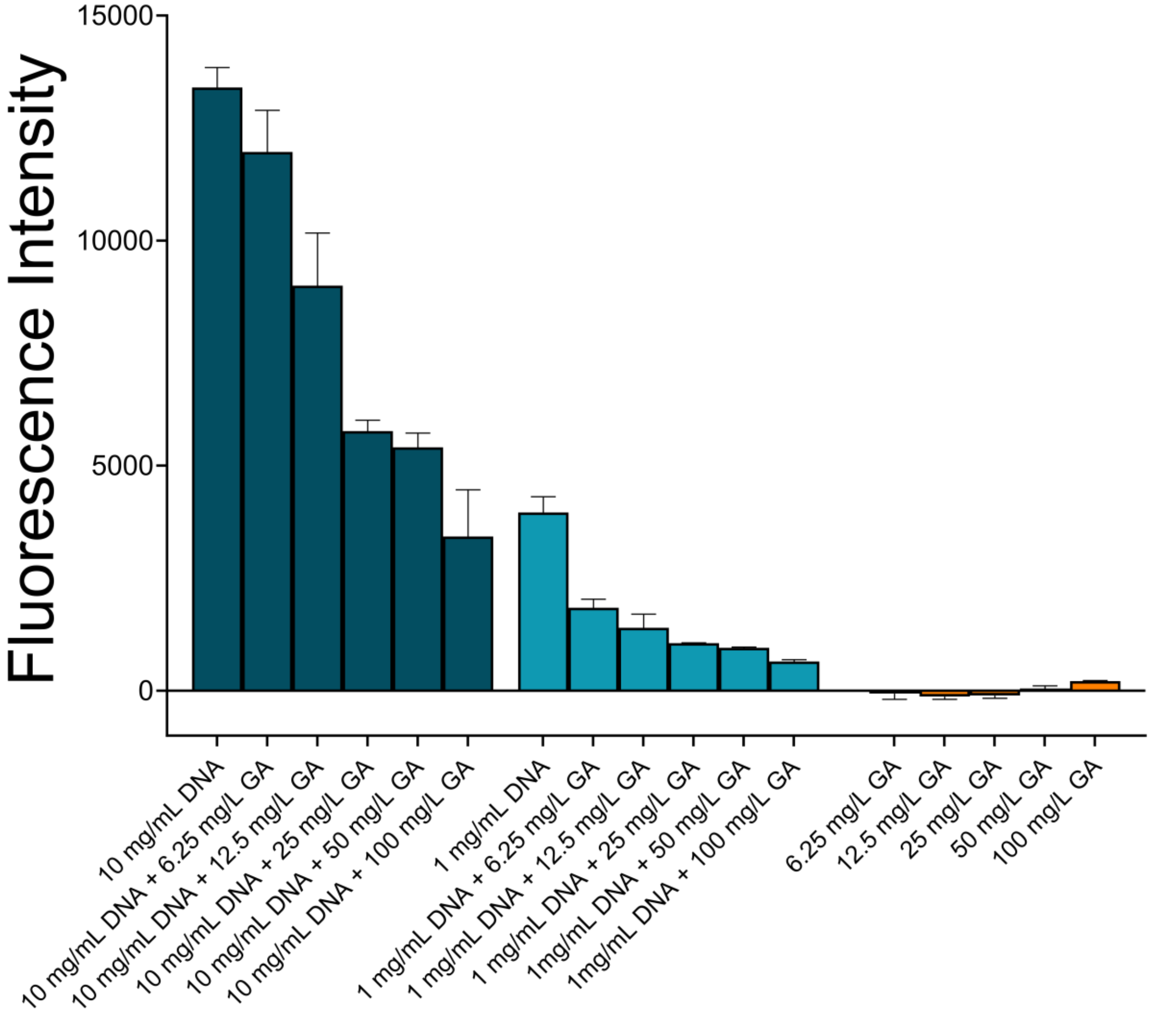

Supplementary Figure 5. Propidium iodide fluorescence of 10 and 1 mg/mL DNA after incubation with increasing concentrations of GA along with those GA concentrations in the absence of GA. Medians with 95%CI. n = 5.

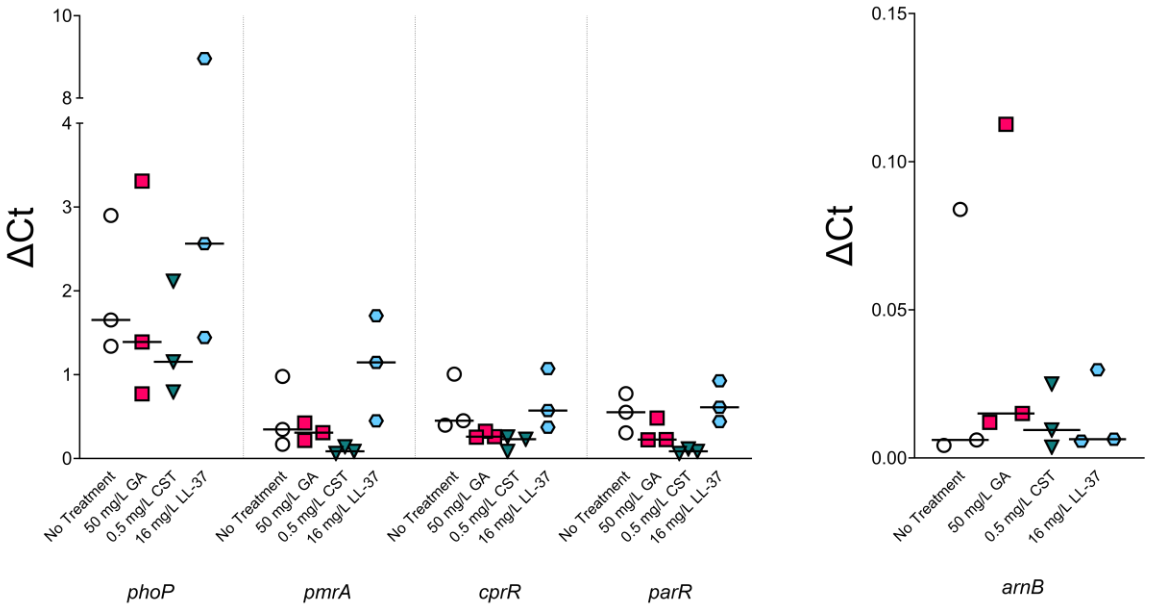

Supplementary Figure 6. Expression of Two Component System genes phoP, pmrA, cprR and parR and L-Ara4N modification gene arnB of P. aeruginosa strains PAO1, PA14 and PAK after exposure to No Treatment or AMPs GA, CST and LL-37 (ΔCt). No significant changes in ΔCt were seen (Friedman test with Dunn's multiple comparison) across the 3 reference strains. Gene expression values can be found in Supplementary Material.

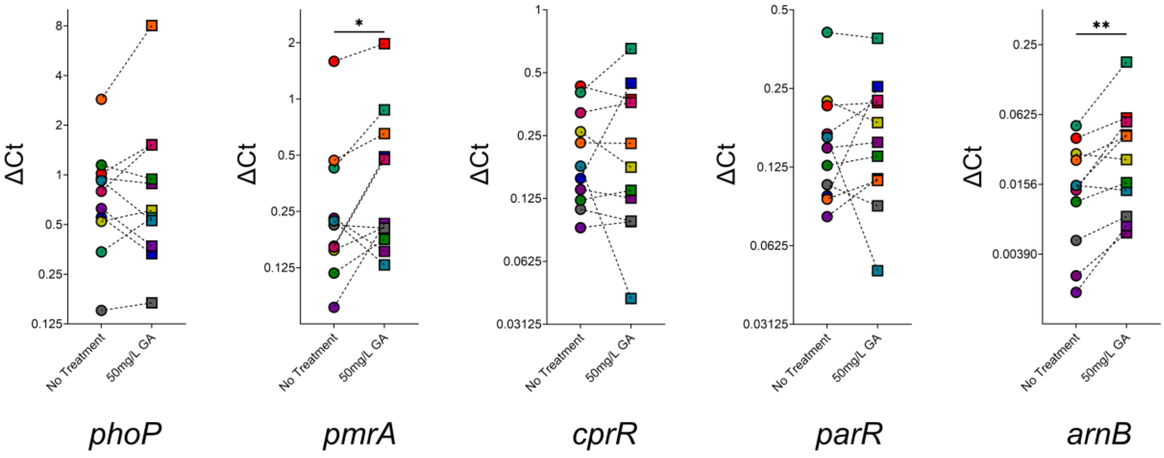

Supplementary Figure 7. Expression of Two Component System genes phoP, pmrA, cprR and parR and L-Ara4N modification gene arnB of P. aeruginosa clinical strains from people with CF after exposure to No Treatment or 50 mg/L GA (ΔCt). GA resulted in significant increases in ΔCt of pmrA (p = 0.042) and anrB (p = 0.0068) from No Treatment (Wilcoxon test. n = 11). Gene expression values can be found in Supplementary Material.

Supplementary Table 1. GenBank Accession Numbers.

	phoP	***phoQ***	***pmrA***	***pmrB***	***cprS***	***cprR***	***parS***	***parR***
**GA_750**	OR023699	OR023710	OR023677	OR023688	OR023633	OR023644	OR023655	OR023666
**GA_899**	OR023700	OR023711	OR023678	OR023689	OR023634	OR023645	OR023656	OR023667
**GA_550**	OR023698	OR023709	OR023676	OR023687	OR023632	OR023643	OR023654	OR023665
**GA_294**	OR023693	OR023704	OR023671	OR023682	OR023627	OR023638	OR023649	OR023660
**GA_982**	OR023701	OR023712	OR023679	OR023690	OR023635	OR023646	OR023657	OR023668
**GA_422**	OR023694	OR023705	OR023672	OR023683	OR023628	OR023639	OR023650	OR023661
**GA_519**	OR023697	OR023708	OR023675	OR023686	OR023631	OR023642	OR023653	OR023664
**GA_461**	OR023695	OR023706	OR023673	OR023684	OR023629	OR023640	OR023651	OR023662
**GA_490**	OR023696	OR023707	OR023674	OR023685	OR023630	OR023641	OR023652	OR023663
**GA_065**	OR023691	OR023702	OR023669	OR023680	OR023625	OR023636	OR023647	OR023658
**GA_072**	OR023692	OR023703	OR023670	OR023681	OR023626	OR023637	OR023648	OR023659

Supplementary Table 2. Clinical P. aeruginosa isolates used in this study.

Strain		Sample Type	Mucoidy	Multidrug Resistance	CST	TOB	GA/TOB Synergy*
**GA_750**		Spontaneous Sputum	Mucoid		S	S	✓
**GA_899**		Spontaneous Sputum			S	S	
**GA_550**		Spontaneous Sputum			S	S	
**GA_294**		Spontaneous Sputum	Mucoid		S	S	
**GA_982**		Spontaneous Sputum			S	S	✓
**GA_422**		Spontaneous Sputum			R	S	✓
**GA_519**		Spontaneous Sputum	Mucoid		S	R	✓
**GA_461**		Cough Swab		MDR	S	R	
**GA_490**		Spontaneous Sputum		MDR	S	R	✓
**GA_065**		Spontaneous Sputum			S	R	
**GA_072**		Spontaneous Sputum			S	R	✓

S – sensitive; R – resistant

MDR – multidrug resistant i.e. resistant to three or more antibiotics from different classes [1,2]

**characterised in Murphy et al., 2022* [3]

Supplementary Table 3. Mass to charge (m/z) ratios of unmodified Native Lipid A and each type of modified Lipid A measured by MALDI-TOF.

	**m/z**
Native Lipid A	1404 1447 1462
Phosphate	1484 1527 1542 1654 1697 1712 1722 1765 1780
C10:30OH	1494 1537 1552 1574 1617 1632 1654 1697 1705 1712 1748 1763 1812 1836 1855 1870 1879 1894
Palmitate	1562 1605 1620 1642 1685 1700 1722 1765 1773 1780 1812 1816 1831 1855 1870 1904 1947 1962
L-Ara4N	1535 1578 1593 1666 1705 1709 1724 1748 1763 1773 1816 1831 1836 1879 1894 1904 1947 1962

1. Santajit, S. & Indrawattana, N. Mechanisms of Antimicrobial Resistance in ESKAPE Pathogens. *Biomed Res. Int.* **2016**, (2016).

2. Magiorakos, A. P. *et al.* Multidrug-resistant, extensively drug-resistant and pandrug-resistant bacteria: An international expert proposal for interim standard definitions for acquired resistance. *Clin. Microbiol. Infect.* **18**, 268–281 (2012).

3. Murphy, R. A. *et al.* Synergistic Activity of Repurposed Peptide Drug Glatiramer Acetate with Tobramycin against Cystic Fibrosis Pseudomonas aeruginosa. *Microbiol. Spectr.* e0081322 (2022) doi:10.1128/spectrum.00813-22.
